# Supplementary material for: Effect of Pemafibrate on Hemorheology in Patients with Hypertriglyceridemia and Aggravated Blood Fluidity Associated with Type 2 Diabetes or Metabolic Syndrome
Source: J Clin Med. 2023 Feb 13;12(4):1481. doi: 10.3390/jcm12041481 (PMC9962113; doi:10.3390/jcm12041481)
Supplement: Supplementary file 1 [file jcm-12-01481-s001.zip › jcm-2077140-supplementary.pdf]

Supplementary Materials:

Supplementary Table S1. Characteristic of hemorheological parameters in participants at week 8.

	Overall (n=96)	Non Pemafibrate (n=46)	Pemafibrate (n=50)	*P* Value
Leukocyte (x10^3/μL)	7.07±1.96	7.49±1.87	6.70±2.00	0.06
RBC (x10^2/μL)	4.82±0.49	4.77±0.46	4.87±0.52	0.38
Hematocrit (%)	43.8±3.6	43.7±3.9	44.0±3.4	0.74
Platelet (x10^4/μL)	235.9±69.4	224.0±66.9	246.6±71.3	0.12
Glucose (mg/dL)	132.3±40.7	130.0±35.1	134.3±45.8	0.62
HbA1c (%)	7.2±1.1	7.2±1.1	7.2±1.3	0.99
CPK (mg/dL)	108.8±56.1	114.9±61.7	103.4±51.4	0.34
AST (mg/dL)	28.5±13.7	26.9±12.4	30.0±14.9	0.28
ALT (mg/dL)	24.0±15.5	28.0±17.8	20.6±12.5	<0.05
γGTP (mg/dL)	43.6±50.7	48.8±43.3	39.0±57.0	0.37

Data are presented as the mean±SD for continuous parameters and % (n) for categorical parameters. Abbreviations: DPP–4, dipeptidyl peptidase–4; CPK, creatin phosphokinase; AST, aspartate aminotransferase; ALT, alanine aminotransferase; GTP, γ–glutamyl transpeptidase

Supplementary Table S2. Characteristic of hemorheological parameters in participants at week 16.

	Overall (n=96)	Non Pemafibrate (n=46)	Pemafibrate (n=50)	*P* Value
Leukocyte (x10^3/μL)	7.00±1.98	7.36±1.81	6.66±2.12	0.09
RBC (x10^2/μL)	4.83±0.52	4.76±0.50	4.89±0.55	0.25
Hematocrit (%)	44.0±3.8	43.7±3.9	44.2±3.7	0.5
Platelet (x10^4/μL)	233.8±70.2	218.4±62.6	248.5±75.2	<0.05
Glucose (mg/dL)	128.6±33.4	135.8±32.8	121.7±33.2	<0.05
HbA1c (%)	7.2±1.1	7.3±1.1	7.0±1.2	0.35
CPK (mg/dL)	117.2±65.3	124.0±70.8	110.7±60.4	0.34
AST (mg/dL)	28.5±13.4	26.5±10.7	30.3±15.6	0.18
ALT (mg/dL)	25.0±16.7	28.1±16.0	22.0±17.1	0.08
γGTP (mg/dL)	46.5±76.0	45.5±36.9	47.4±101.2	0.9

Data are presented as the mean±SD for continuous parameters and % (n) for categorical parameters. Abbreviations: DPP–4, dipeptidyl peptidase–4; CPK, creatin phosphokinase; AST, aspartate aminotransferase; ALT, alanine aminotransferase; GTP, γ–glutamyl transpeptidase

Supplementary Table S3. Characteristic of hemorheological parameters in participants at week 8.

	Overall (n=96)	Non Pemafibrate (N=46)	Pemafibrate (n=50)	*P* Value
Total cholesterol (mg/dL)	173.3±29.8	171.5±29.2	175.0±30.8	0.58
Low–Density Lipoprotein cholesterol (mg/dL)	90.5±27.8	85.6±26.1	94.9±29.2	0.12
Triglyceride (mg/dL)	182.6±121.5	205.9±139.6	161.8±101.1	0.09
High–Density Lipoprotein cholesterol (mg/dL)	46.4±10.6	44.9±11.3	47.7±10.1	0.21
RLP (mg/dL)	7.6±7.2	8.8±9.5	5.5±3.1	<0.01
hsCRP (mg/dL)	0.31±0.96	0.22±0.26	0.39±1.31	0.43
BAP (μmol/L)	2292.2±433.8	2264±397.1	2318.1±471.9	0.56
d–ROMs (U.CARR)	334.7±70.4	367.3±57.3	304.8±69.8	<0.01
FFA (mEq/L)	0.69±0.31	0.75±0.30	0.63±0.31	0.06
Whole blood transit time (s)	71.5±43.6	66.3±37.8	76.1±48.7	0.29
Number of adhesive leukocytes (/HPF)	12.5±7.6	12.14±7.07	12.8±8.25	0.68

Data are presented as the mean±SD for continuous parameters and % (n) for categorical parameters. Abbreviations: RLP–C, remnant like particles cholesterol; hsCRP, high sensitivity C–reactive protein; BAP, biological antioxidant potential; d–ROMs, diacron reactive oxygen metabolites; FFA, free fatty acid

Supplementary Table S4. Characteristic of hemorheological parameters in participants at week 16.

	Overall (n=96)	Non Pemafibrate (N=46)	Pemafibrate (n=50)	*P* Value
Total cholesterol (mg/dL)	171.5±29.4	171.5±31.0	171.5±28.5	0.99
Low–Density Lipoprotein cholesterol (mg/dL)	89.1±27.5	81.9±27.3	95.9±26.6	<0.05
Triglyceride (mg/dL)	177.1±116.5	220.5±148.6	135.5±49.1	<0.01
High–Density Lipoprotein cholesterol (mg/dL)	46.5±11.2	44.6±11.5	48.4±10.9	0.12
RLP (mg/dL)	7.3±7.3	9.9±9.6	4.9±2.5	<0.01
hsCRP (mg/dL)	0.235±0.463	0.207±0.277	0.262±0.595	0.58
BAP (μmol/L)	2377±410.3	2338.6±459.2	2412.4±367.6	0.40
d–ROMs (U.CARR)	338.7±63.6	362.6±50.8	317.3±67.4	<0.01
FFA (mEq/L)	0.69±0.26	0.72±0.31	0.65±0.21	0.19
Whole blood transit time (s)	63.4±34.8	68.0±42.0	59.2±27.0	0.22
Number of adhesive leukocytes (/HPF)	12.05±7.50	12.04±7.31	12.06±7.81	0.99

Data are presented as the mean±SD for continuous parameters and % (n) for categorical parameters. Abbreviations: RLP–C, remnant like particles cholesterol; hsCRP, high sensitivity C–reactive protein; BAP, biological antioxidant potential; d–ROMs, diacron reactive oxygen metabolites; FFA, free fatty acid
